# Birth order and sickness absence: Register-based evidence from Finland

**DOI:** 10.1371/journal.pone.0280532

**Published:** 2023-01-17

**Authors:** Kaarina Reini, Jan Saarela

**Affiliations:** Demography Unit, Faculty of Education and Welfare Studies, Åbo Akademi University, Vaasa, Finland; Girne American University - Karmi Campus: Girne Amerikan Universitesi, CYPRUS

## Abstract

**Background:**

In working ages, sickness absence is strongly related to persons’ health condition. We studied how birth order was associated with receipt of sickness allowance, distinguishing between mental disorders, musculoskeletal disorders and injuries.

**Methods:**

A follow-up study based on the entire Finnish population was conducted for sibling groups born 1969–1982, in which each sibling was observed from age 35 years in the period 2004–2018. Focus was on within-family variation in first-time sickness allowance receipt.

**Results:**

Results of stratified Cox regressions revealed that each increase in birth order was associated with a slightly higher risk of sickness absence from any cause. For mental disorders, associations were stronger; the hazard ratio as compared to first borns was 1.03 (95% CI: 0.98–1.08) of second borns, 1.10 (0.99–1.22) of third borns, and 1.52 (1.25–1.85) of fourth or higher borns. Corresponding numbers for musculoskeletal disorders were 1.12 (1.07–1.17), 1.19 (1.09–1.30) and 1.15 (0.96–1.38), and for injuries 1.06 (1.01–1.12), 1.09 (1.21–1.14) and 0.96 (0.77–1.20), respectively.

**Conclusions:**

Birth order effects were generally stronger for women than men, and to some extent influenced by educational level, occupation, income, and family composition. Possible latent mechanisms behind the associations may relate to within-family dynamics at childhood.

## Introduction

Analyses of the interrelation between birth order and various health outcomes have found that later born siblings generally perform worse than earlier born siblings. This includes depression, mental distress, psychiatric deviation, anxiety, self-esteem, physical fitness and hospitalization for alcohol use [1–4]. Higher birth order has been found related also to higher mortality from external causes, primarily suicides, but also accidents and events of undetermined event, and to alcohol-related mortality [5–9].

Similar to studies on educational attainment, intelligence, and cognitive and non-cognitive skills [10–13], a number of potential explanations to the role of birth order have been suggested. Siblings may part of a dynamic environment that become less cognitively stimulating when the family grows in size, parental resources may decrease, the likelihood of communicable diseases may increase, and there may be biological depletion of mothers with additional births [14–19]. Another set of explanations relate to the social environment within the family. Siblings may occupy different niches to avoid competition, and there can be bullying at the expense of later borns [20, 21]. A particularly influential theory has emphasized how the within-family dynamics create more rebellious later-born children [22], which may be expressed through higher risk taking that affect health and premature mortality.

A fundamental contribution of recent empirical studies [3–9] is the use of family fixed effects methods. They study variation within families only, and not variation between families. This approach minimises residual confounding from unmeasured time-invariant factors that are shared amongst siblings, such as common genetic factors, parenting style, socioeconomic environment at childhood, and parental health behaviours. Using sibling fixed effects, this study is the first to analyse if sickness absence by main cause is associated with birth order in prime working ages.

In working ages, sickness absence is strongly related to a person’s health status [23, 24]. Sickness absence has been linked to all cause mortality [24, 25] and to mortality from cardiovascular disease, cancer, alcohol related causes and suicide [25]. Medically certified sickness absence is also a more powerful predictor of mortality than poor self reported health and other objective measures of specific medical conditions [24]. In addition, a long sickness absence predicts disability retirement in various occupations [26]. Based on previous studies of how birth order associates to mental and physical health outcomes, and accidents, we expect, therefore, that it should be associated with sickness absence from mental disorders, musculoskeletal disorders, and injuries.

The multigenerational population register data used were based on the entire Finnish population. Sickness absence was measured by receipt of sickness allowance (SA), to which all non-retired persons are eligible after the ninth sickness day. Diagnoses were distinguished by the tenth revision of International Classification of Diseases (ICD-10). By constructing complete sibling groups, we sought to study the association between birth order and sickness absence from main cause in the working-aged Finnish population. In particular, we analysed whether any association between birth order and sickness absence manifests in mental disorders, musculoskeletal disorders, and injuries, respectively, and whether it depends on sex, mother’s age at birth, educational level, occupation, income, and family composition.

## Materials and methods

We used data from the Finnish population register, which contains the entire population resident in the Finland. All data access, data preparation and analyses were performed within Statistics Finland’s remote access system Fiona, with contract number TK-52-694-18. Statistics Finland’s guidelines for handling data were followed. According to the Statistics Act in Finland, Statistics Finland decides independently on all licenses that are granted for use of data [27]. The data used in the study can be obtained from Statistics Finland by other researchers, conditional on that they are granted access and pay the service fees. All data were fully anonymized before we accessed them. Since they were register-based and anonymized, there was no need to seek separate ethical approval from an ethnic committee for the study, and no need to obtain consent from the study persons.

Each person born in Finland can be linked to his or her mother and father, subject to that the parent ever had resided in Finland. We constructed, therefore, sibling groups based on the native-born population. These multigenerational data were linked to registries on births, deaths, migration, educational attainment, occupation, income, marriages and cohabitation. Each person was linked also to data on SA receipt from the Social Insurance Institution of Finland (KELA), covering the period 2004–2018. All non-retired persons aged 16–67 years are eligible for SA, which compensates for work incapacity. SA is paid after a waiting period of nine working days, and for a maximum period of approximately one calendar year. If the person remains in work incapacity thereafter, he or she may apply for disability pension. SA receipt is conditional on a physician’s certificate that includes the main diagnosis for work incapacity according to ICD-10. Receipt of SA is consequently a highly reliable and nationally covering objective proxy for sickness beyond a conventional flu. There is no national register on shorter sickness absence spells in Finland.

Diagnoses were categorized into the three most common groups in Finland: mental disorders (F00-F99), musculoskeletal disorders (M00-M99), and injuries (S00 and T98). All remaining causes were classified into a residual group.

To ensure that all siblings in a sibling group were observed from the same age in the study period 2004–2018, we restricted analyses to persons born 1969–1982. Each person was observed from age 35 years until first SA receipt, death, emigration, or end of 2018, whichever came first. Data on SA receipt with diagnosis information was not available before 2004. This implies that the study persons could have been on SA before age 35. Starting from age 35 was because the control variables should have a meaningful interpretation. By age 35, most people have completed their education, entered a profession, stabilized their income level, and formed a family. All study persons were registered as Finnish or Swedish speakers.

To analyse the association between birth order and SA receipt, we applied Cox proportional hazards models with family fixed effects. In these models, siblings share the same baseline hazard. We ran parallel models in which first-time SA receipt due to mental disorders, musculoskeletal disorders, injuries, and other causes, respectively, constituted the failure event. The baseline hazard for SA receipt was time since age 35. Right-censoring occurred at death, emigration, end of the observation period, or SA receipt due to a main cause other than the studied failure event. By comparing siblings in the same family, we effectively adjusted for all time invariant observed and unobserved factors that are shared by siblings within the same family. This approach can be considered superior to the regular Cox proportional hazard model, because it minimises confounding from unobserved or unmeasured time invariant variables. A sibling group refers to the shared mother id plus father id, meaning that we analyse full-sibling groups. A requirement for the analyses is that there must be at least two siblings in the group, and at least one must have received SA due to each set of ICD-10 codes utilised in each of the parallel models.

We adjusted for each sibling’s birth year, sex, mother’s age when giving birth, educational level, occupation, income quintile, and family composition, which are known health correlates. All variables were categorised, and introduced into the models in a stepwise manner. Father’s age at child birth was excluded due to lower explanatory power than mother’s age at birth. There was no need to adjust for parental factors like mother’s or father’s education or socioeconomic status, because the sibling comparison removes the confounding effect of time invariant factors shared by the siblings. We also conducted analyses where birth order and sex were interacted, in order to evaluate any sex differences on this concern. Estimates are presented as hazard ratios (HRs) with 95% confidence intervals (95% CIs). First borns serve as the reference category. All estimations were carried out with R version 3.6.3.

## Results

Characteristics of the study population are presented in Table 1. Among 318,021 siblings in 146,604 siblings groups, there were in total 124,917 persons who received SA from any cause. The number of first-time SA recipients by each main cause, that is, mental disorders, musculoskeletal disorders, injuries, or other causes, were 24,069, 30,155, 22,678, and 48,015, respectively.

**Table 1:** Descriptive statistics of the study population.

Birth order (%)	
1st	45.9
2nd	45.0
3rd	8.0
4th or higher	1.1
Sex (%)	
Man	51.3
Woman	48.7
Birth year (%)	
1969–1972	24.9
1973–1975	25.3
1976–1978	25.5
1979–1982	24.4
Mother’s age at birth (%)	
-20 years	9.3
21–25 years	36.3
26–30 years	36.0
31–35 years	15.0
36+ years	3.5
Educational level (%)	
Primary	0.8
Secondary	47.7
Tertiary	51.5
Occupation (%)	
Managers	3.5
Professionals	21.1
Technicians and associate professionals	18.5
Clerical support workers	4.9
Service and sales workers	13.6
Skilled agricultural, forestry and fishery workers	1.8
Craft and related trade workers	8.3
Plant and machine operators and assemblers	7.4
Elementary occupations	4.5
No occupation or unknown	16.5
Income quintile (%)	
1st	20.0
2nd	20.0
3rd	20.0
4th	20.0
5th	20.0
Family composition (%)	
With partner and children	59.8
With partner, no children	14.8
Single	17.8
Other	7.6
Number of SA recipients by	
Mental disorder	24,069
Musculoskeletal disorder	30,155
Injury	22,678
Other cause	48,015
Number of sibling groups	146,604
Number of siblings	318,021
Number of person years	1,916,587

The description refers to the complete cohorts, i.e., full-sibling groups with at least two siblings, in which all siblings were born 1969–1982, observed in 2004–2018, and followed from age 35.

Numbers used in the stratified Cox regressions are provided in Table 2.

Mental disorders refer to ICD-10 codes F00-F99, musculoskeletal disorders to M00-M99, injuries to S00-T98, and other causes to all other codes.

The stratified Cox regressions revealed that birth order was associated with a slightly higher risk of SA receipt from any cause (Table 2). As compared with first borns, the HRs of second borns, third borns, and fourth or higher borns, respectively, were 1.04 (95% CI: 1.01, 1.06), 1.05 (1.01–1.10), and 1.07 (0.97–1.17). This association for all cause SA receipt was partly related to educational attainment, occupation and income differences across birth order. In the model that adjusted for all confounders, there were no significant differences in all cause SA receipt by birth order.

**Table 2:** Hazard ratios with 95% confidence intervals for estimated associations between birth order and sickness absence, results from Cox regression models stratified by shared mother and father identification.

Cause of SA receipt	Model 1		Model 2		Model 3		Model 4		Model 5		Model 6	
by birth order	HR	95% CI	HR	95% CI	HR	95% CI	HR	95% CI	HR	95% CI	HR	95% CI
All causes												
1st	1		1		1		1		1		1	
2nd	1.04	1.01–1.06	1.04	1.02–1.07	1.03	1.01–1.05	1.02	0.99–1.04	1.02	0.99–1.04	1.02	0.99–1.04
3rd	1.05	1.01–1.10	1.07	1.02–1.12	1.05	1.00–1.10	1.04	0.99–1.09	1.03	0.99–1.08	1.03	0.98–1.08
4th or higher	1.07	0.97–1.17	1.09	0.99–1.20	1.06	0.97–1.16	1.04	0.95–1.14	1.03	0.94–1.13	1.03	0.94–1.13
Number of SA recipients	76,507											
Number of sibling groups	68,293											
Number of siblings	156,754											
Number of person years	904,872											
Mental disorders												
1st	1		1		1		1		1		1	
2nd	1.03	0.98–1.08	1.03	0.97–1.08	1.02	0.97–1.08	1.02	0.96–1.07	1.02	0.96–1.07	1.00	0.95–1.06
3rd	1.10	0.99–1.22	1.08	0.97–1.20	1.08	0.97–1.20	1.07	0.96–1.19	1.07	0.96–1.19	1.05	0.95–1.17
4th or higher	1.52	1.25–1.85	1.50	1.23–1.83	1.49	1.22–1.82	1.48	1.21–1.81	1.48	1.21–1.81	1.46	1.19–1.79
Number of SA recipients	21,719											
Number of sibling groups	21,127											
Number of siblings	48,602											
Number of person years	242,463											
Musculoskeletal disorders												
1st	1		1		1		1		1		1	
2nd	1.12	1.07–1.17	1.13	1.08–1.18	1.10	1.05–1.15	1.08	1.03–1.13	1.08	1.03–1.13	1.08	1.03–1.13
3rd	1.19	1.09–1.30	1.22	1.11–1.34	1.17	1.06–1.28	1.15	1.04–1.26	1.14	1.03–1.25	1.14	1.03–1.25
4th or higher	1.15	0.96–1.38	1.20	0.99–1.44	1.13	0.94–1.36	1.11	0.92–1.34	1.09	0.90–1.32	1.09	0.90–1.32
Number of SA recipients	26,683											
Number of sibling groups	25,726											
Number of siblings	59,256											
Number of person years	296,686											
Injuries												
1st	1		1		1		1		1		1	
2nd	1.06	1.01–1.12	1.09	1.03–1.15	1.07	1.01–1.13	1.06	1.00–1.12	1.06	1.00–1.12	1.05	0.99–1.11
3rd	1.09	0.98–1.21	1.14	1.02–1.27	1.11	0.99–1.23	1.09	0.98–1.22	1.09	0.97–1.22	1.09	0.97–1.21
4th or higher	0.96	0.77–1.20	1.03	0.82–1.28	0.98	0.78–1.22	0.96	0.77–1.21	0.96	0.76–1.20	0.95	0.76–1.19
Number of SA recipients	20,842											
Number of sibling groups	20,352											
Number of siblings	46,706											
Number of person years	238,637											
Other causes												
1st	1		1		1		1		1		1	
2nd	0.97	0.94–1.00	0.98	0.94–1.00	0.97	0.93–1.00	0.96	0.93–0.99	0.96	0.92–0.99	0.96	0.93–0.99
3rd	0.93	0.86–1.00	0.94	0.87–1.02	0.93	0.86–1.00	0.93	0.86–0.99	0.92	0.85–0.99	0.92	0.85–0.99
4th or higher	0.86	0.74–1.00	0.87	0.75–1.02	0.86	0.73–0.99	0.84	0.72–0.98	0.84	0.72–0.98	0.84	0.72–0.98
Number of SA recipients	40,946											
Number of sibling groups	39,141											
Number of siblings	89,950											
Number of person years	470,759											

Model 1 adjusts for Birth order, Sex and Birth year. Model 2 adjusts for variables in Model 1 plus Motherʾs age at birth. Model 3 adjusts for variables in Model 2 plus Educational level. Model 4 adjusts for variables in Model 3 plus Occupation. Model 5 adjusts for variables in Model 4 plus Income quintile. Model 6 adjusts for variables in Model 5 plus Family composition.

Birth order was associated with SA receipt from mental disorders, musculoskeletal disorders, and to some extent injuries, but not with other causes (Table 2). As compared to first borns, the HR for SA receipt due to mental disorders was 1.03 (0.98–1.08) of second borns, 1.10 (0.99–1.22) of third borns, and 1.52 (1.25–1.85) of fourth or higher borns. Corresponding numbers for musculoskeletal disorders were 1.12 (1.07–1.17), 1.19 (1.09–1.30), and 1.15 (0.96–1.38), and for injuries 1.06 (1.01–1.12), 1.09 (0.98–1.21), and 0.96 (0.77–1.20). These associations were to some extent explained by differences in education, occupation and income.

Models that interacted birth order with sex revealed that the associations between birth order and SA receipt were notably stronger for women than men (Table 3). As compared with first-born women, the HR for SA receipt due to mental disorders was 1.07 (1.01–1.14) of second-born women, 1.19 (1.06–1.34) of third-born women, and 1.50 (1.19–1.90) of fourth- or higher born women. Corresponding numbers for SA receipt due to musculoskeletal disorders in women were 1.11 (1.05–1.17), 1.20 (1.08–1.34), and 1.32 (1.04–1.66). For men, the pattern was less consistent. As compared with first-born men, the HR for SA receipt due to mental disorders was 0.98 (0.91–1.05) of second-born men, 0.97 (0.85–1.11) of third-born men, and 1.56 (1.19–2.04) of fourth- or higher born men. Corresponding numbers for SA receipt due to musculoskeletal disorders in men were 1.12 (1.06–1.19), 1.18 (1.05–1.31), and 0.99 (0.77–1.27). SA receipt due to injuries displayed a more clear divide between first and later borns in women as compared to men. For SA receipt due to other causes there were no associations with birth order for either sex.

**Table 3:** Hazard ratios with 95% confidence intervals for estimated associations of birth order and sickness absence by sex, results from Cox regression models stratified by shared mother and father identification.

Cause of SA receipt	Model 1		Model 2		Model 3		Model 4		Model 5		Model 6	
by birth order	HR	95% CI	HR	95% CI	HR	95% CI	HR	95% CI	HR	95% CI	HR	95% CI
Men, All causes												
1st	1		1		1		1		1		1	
2nd	1.02	0.99–1.05	1.03	0.99–1.06	1.01	0.98–1.04	1.01	0.98–1.04	1.00	0.97–1.03	1.00	0.97–1.03
3rd	1.05	0.99–1.11	1.07	1.01–1.14	1.05	0.99–1.11	1.05	0.99–1.11	1.04	0.98–1.10	1.04	0.98–1.10
4th or higher	0.96	0.85–1.09	0.99	0.87–1.12	0.96	0.85–1.09	0.95	0.84–1.08	0.94	0.83–1.06	0.93	0.82–1.06
Men, Mental disorders												
1st	1		1		1		1		1		1	
2nd	0.98	0.91–1.05	0.97	0.90–1.05	0.97	0.90–1.05	0.97	0.90–1.05	0.97	0.90–1.04	0.95	0.89–1.03
3rd	0.97	0.85–1.11	0.96	0.83–1.10	0.96	0.83–1.10	0.96	0.83–1.11	0.96	0.83–1.10	0.94	0.81–1.09
4th or higher	1.56	1.19–2.04	1.53	1.17–2.01	1.54	1.18–2.03	1.56	1.18–2.05	1.55	1.18–2.04	1.52	1.15–2.00
Men, Musculoskeletal disorders												
1st	1		1		1		1		1		1	
2nd	1.12	1.06–1.19	1.14	1.07–1.21	1.10	1.04–1.17	1.10	1.03–1.17	1.09	1.03–1.16	1.09	1.03–1.16
3rd	1.18	1.05–1.31	1.21	1.08–1.35	1.16	1.04–1.31	1.16	1.03–1.30	1.15	1.02–1.29	1.15	1.02–1.29
4th or higher	0.99	0.77–1.27	1.04	0.81–1.33	1.00	0.77–1.28	0.99	0.77–1.28	0.97	0.75–1.25	0.97	0.75–1.25
Men, Injuries												
1st	1		1		1		1		1		1	
2nd	0.99	0.93–1.06	1.02	0.95–1.09	1.00	0.93–1.06	0.99	0.92–1.06	0.99	0.92–1.05	0.98	0.92–1.05
3rd	1.12	0.99–1.26	1.17	1.03–1.33	1.13	0.99–1.28	1.12	0.98–1.27	1.11	0.98–1.26	1.11	0.98–1.26
4th or higher	0.88	0.67–1.16	0.94	0.72–1.24	0.90	0.68–1.18	0.88	0.67–1.17	0.87	0.66–1.16	0.87	0.66–1.15
Men, Other causes												
1st	1		1		1		1		1		1	
2nd	0.97	0.92–1.02	0.97	0.93–1.03	0.97	0.92–1.02	0.96	0.91–1.01	0.96	0.91–1.01	0.96	0.91–1.01
3rd	0.98	0.89–1.08	0.99	0.90–1.10	0.98	0.89–1.08	0.98	0.89–1.08	0.98	0.88–1.08	0.98	0.88–1.08
4th or higher	0.78	0.62–0.97	0.79	0.63–0.99	0.78	0.62–0.97	0.76	0.61–0.96	0.75	0.60–0.95	0.76	0.60–0.95
Women, All causes												
1st	1		1		1		1		1		1	
2nd	1.05	1.02–1.08	1.06	1.03–1.09	1.04	1.01–1.08	1.03	1.01–1.07	1.03	1.00–1.06	1.03	1.00–1.06
3rd	1.05	1.00–1.11	1.07	1.01–1.13	1.05	0.99–1.11	1.03	0.98–1.09	1.03	0.97–1.09	1.03	0.97–1.08
4th or higher	1.16	1.04–1.30	1.19	1.06–1.33	1.15	1.02–1.29	1.13	1.00–1.26	1.12	0.99–1.26	1.11	0.99–1.25
Women, Mental disorders												
1st	1		1		1		1		1		1	
2nd	1.07	1.01–1.14	1.06	0.99–1.14	1.06	0.99–1.13	1.05	0.98–1.12	1.05	0.98–1.12	1.04	0.97–1.11
3rd	1.19	1.06–1.34	1.18	1.04–1.33	1.17	1.04–1.32	1.15	1.02–1.30	1.15	1.02–1.30	1.13	1.00–1.28
4th or higher	1.50	1.19–1.90	1.47	1.16–1.87	1.46	1.15–1.86	1.44	1.13–1.83	1.44	1.13–1.83	1.42	1.11–1.80
Women, Musculoskeletal disorders												
1st	1		1		1		1		1		1	
2nd	1.11	1.05–1.17	1.12	1.06–1.19	1.09	1.03–1.16	1.07	1.00–1.13	1.06	1.00–1.13	1.06	1.00–1.13
3rd	1.20	1.08–1.34	1.23	1.10–1.38	1.17	1.04–1.31	1.13	1.01–1.27	1.12	0.99–1.26	1.12	0.99–1.26
4th or higher	1.32	1.04–1.66	1.37	1.08–1.74	1.27	1.00–1.61	1.23	0.97–1.57	1.22	0.96–1.56	1.22	0.96–1.55
Women, Injuries												
1st	1		1		1		1		1		1	
2nd	1.17	1.08–1.25	1.19	1.11–1.29	1.17	1.09–1.26	1.16	1.08–1.25	1.16	1.07–1.25	1.15	1.07–1.25
3rd	1.04	0.90–1.20	1.09	0.94–1.26	1.06	0.91–1.22	1.05	0.90–1.21	1.04	0.90–1.21	1.04	0.90–1.21
4th or higher	1.08	0.79–1.47	1.15	0.84–1.57	1.10	0.80–1.50	1.08	0.79–1.49	1.07	0.78–1.47	1.07	0.78–1.47
Women, Other causes												
1st	1		1		1		1		1		1	
2nd	0.97	0.93–1.01	0.98	0.93–1.02	0.97	0.92–1.01	0.96	0.92–1.01	0.96	0.92–1.00	0.96	0.92–1.01
3rd	0.89	0.82–0.97	0.90	0.83–0.99	0.89	0.82–0.97	0.89	0.81–0.97	0.88	0.80–0.96	0.88	0.81–0.96
4th or higher	0.92	0.76–1.10	0.93	0.77–1.12	0.91	0.76–1.10	0.90	0.75–1.08	0.89	0.74–1.08	0.90	0.74–1.08

The models contain same variables as reported in Table 2, except that Birth order and Sex have been substituted with a joint variable for Birth order and Sex. Reported is the association between birth order and sickness absence for men and women, respectively, where we have switched the reference group from first born men to first born women within each model. Numbers of SA recipients, sibling groups, siblings, and person years are consequently the same as reported in Table 2.

For mental disorders in women, and musculoskeletal disorders in men, significant associations with birth order remained also when we controlled for socioeconomic and demographic variables (Table 3). According to the fully adjusted model, second-born women had a HR of SA receipt due to mental disorders that was 1.04 (0.97–1.11) that of first born, third born had a HR of 1.13 (1.00–1.28), and fourth or higher born 1.42 (1.11–1.80). Second-born men had a HR of SA receipt due to musculoskeletal disorders that was 1.09 (1.03–1.16) that of first borns, second-born men had a HR of 1.15 (1.02–1.29), while that of fourth or higher born was 0.97 (0.75–1.25), when birth year, mother’s age at birth, educational level, occupation, income and family composition were controlled for.

Results were largely similar across sibling group size and birth spacing (S1 and S2 Tables).

## Discussion

This study is the first to provide evidence about an interrelation between birth order and sickness absence, utilising diagnose information and family fixed effects methods. We have estimated the risk of first-time SA receipt due to mental disorders, musculoskeletal disorders and injuries after age 35. A clear birth order pattern was observed for these main causes, but not for a residual group that consisted of other miscellaneous causes. Higher birth order was found associated with a higher risk of sickness absence, and particularly so for mental disorders and musculoskeletal disorders in women.

To give a sense of the magnitude of the observed birth order effects on SA receipt, one may compare with the HRs by educational attainment from the same regressions. The birth order difference in the HR between third and first borns for all cause SA, for instance, is almost equal in size to the difference in the HR between secondary and primary educational levels. With regard to SA receipt due to mental disorders, the HR between fourth or higher borns and first borns is roughly of the same size as the difference in the HR between tertiary and primary educational levels. The effect sizes for birth order are thus not negligible.

These findings, which are based on the total population of Finland, corroborate results from neighbouring countries, which also have applied family fixed effects methods to study the interrelation between birth order and health related outcomes. In Sweden, higher birth order has been found associated with an increased risk of all-cause sickness absence, but there has not been any study using data on the underlying cause [28]. Later borns in Sweden have been found shorter than earlier borns [29], which is noteworthy, because height is a strong health predictor. The mortality risk of adult Swedes is also positively associated with birth order, and more so for women than men [7]. In Denmark, first borns have been found less healthy at birth and in the first years of life, but healthier in adolescence, and later borns are more likely to suffer from injuries throughout childhood [30].

In terms of any interrelation with health behaviours, the picture is complex. First borns in Norway have been found more likely to have weight problems, high blood pressure and high triglycerides, but less likely to smoke daily, and more likely to report good physical and mental health [31]. In Sweden, later borns are hospitalized for alcohol and narcotics use at a higher rate than first borns [4]. In Finland, a positive association has been found between birth order and alcohol related mortality, but only for ethnic male Finnish speakers [9].

With regard to suicide, the pattern is uniform across the Nordic countries. In Finland, Sweden and Norway the suicide risk in the general population increases with birth order, with about the same magnitude in all three countries, and the associations are slightly stronger for men than women [5, 6, 8]. Suicide rates are higher in men than in women [32], but women report higher rates of minor psychiatric morbidity than men [33]. We interpret our findings as providing the mirror image, as we have been concerned with sickness absence due to mental disorders, which is a proxy for also less severe mental problems than those leading to suicide.

Some results that may be at least partly contradictory to ours have nevertheless been reported. A study from Great Britain [34] found no evidence for an association between birth order and midlife psychological distress in either men or women. A previous study from Finland found an elevated risk of schizophrenia, which is a severe mental illness, in first borns as compared with later borns, but also an increased risk associated with having a sibling who was less than five years older [35].

Evidence that support biological mechanisms as drivers of birth order associations, and thus potentially the pattern observed here for musculoskeletal disorders, is not conclusive. This is primarily because effects may operate in different directions. Later borns come from older mothers who have higher incidence of chromosomal and congenital defects [36, 37]. Several births can also cause biological depletion of mothers [38, 39]. Closely spaced pregnancies may not allow sufficient time for the mother to restore her depleted nutrient stores, which may reduce her ability to provide a favourable growth environment for the fetus and later sufficient breast milk production for the baby. However, later borns may have also have better in-utero environment, because they generally have an advantage in birth weight [30]. We did not find any clear differences in the birth order associations by birth spacing or sibling group size. Whether the associations observed here, between birth order and sickness absence from musculoskeletal disorders and mental health causes, respectively, are related to biological mechanisms requires further investigation. However, it is known that mechanisms acting early in life can influence sickness absence due to musculoskeletal disorders later in life [40]. In addition, in-utero exposure to maternal stress seems to be associated with increased take-up of AHDH medications during childhood as well as anti-anxiety and depression medications in adulthood [41].

In our analyses, we have adjusted for some individual-level variables that may confound the relationship between birth order and health, such as educational attainment, occupational status, income, and family composition. Birth order is known to be associated with educational attainment [10, 12] but also occupational choice [42]. In our analyses, the influence of education, occupation and income quintile on the association between birth order and sickness allowance receipt was relatively modest. The inclusion of family composition did not affect the results to any noteworthy degree. One may view the variables linked to socioeconomic status as reflecting cognitive abilities, whereas family composition is more reflective of non-cognitive abilities and thus social skills. The social environment in the family home at childhood, and inter-sibling behaviours, may therefore play a role for the association between birth order and SA receipt due to mental disorders that we have observed, and in particular the large difference for mental disorders when comparing fourth or higher borns with first borns. Although our study subjects are of an age when most persons have established a family of their own, and this family influence health behaviours, any effects from the childhood family may not necessarily be offset or reversed. This might perhaps also explain why the birth order effects were larger for women than for men. Women have a greater tendency than men to root their social role within the family and circles of friends, and may therefore be more sensible to relative disadvantage within the family [7].

However, it needs to be stressed that, for the control variables used here, except sex and birth year, causality may be disputed. A person’s health condition may affect his or her educational attainment, occupational position, income, and family composition, and not just only the other way around. Further research is needed to uncover the underlying causal mechanisms behind birth order and health, and presumably with other data and methods than those used here. Parental health and parental socioeconomic status may nevertheless largely be excluded as potential mechanisms, as we have effectively controlled for time-invariant family characteristics with the family fixed effects models. Possible reasons behind the birth order associations might instead stem from other aspects of family dynamics. Older siblings may bully younger ones, with long-term health impacts [21]. Sibling bullying has also been shown to be a potential risk factor for mental health problems and self-harm [43, 44]. Another related reason may be that later-born children feel insecure because of diluted parental resources, in that resource allocation to each child declines with additional children [45]. Mothers tend to spend less time spent in affectionate attachment with later borns as compared with first borns, and this difference appears to be particularly marked if later borns are female [46]. First borns are also generally favoured in terms of the amount of quality time that children have with their parents [47]. Other factors that may contribute are early prenatal and postnatal parental behaviours, in terms of systematic differences by birth order with regard to health counselling, alcohol consumption, smoking or breastfeeding [48, 49].

We have advanced the research field in some relevant respects. Apart from mortality studies, few papers have estimated the associations between birth order and health outcomes in prime working ages using sibling fixed effects [50]. Moreover, a large part of the literature has focused on mental health at younger ages [e.g. 4, 5, 51–53], and thus largely ignored prime working ages. Perhaps most important to stress is that our study has used total population register data to identify siblings, and to measure health status in an objective manner with diagnoses for the sickness absence. The use of register-based data has several strengths. Data are free from memory flaws and selective reporting. Non-participation is avoided, which is often present in surveys that can be affected by reachability, motivation, or ability of respondents. Research has shown that survey participants have less sickness absence than non-participants, which lead to biased results, and particularly so for long-term sickness absence [54, 55].

The study comes also with some important limitations. One is that SA is not a precise proxy for health, but it reflects also how work ability and poor health status match. Depending on the specific characteristics of the job, some people may be able to work although they are experiencing health problems, while some others may receive SA even though they might be capable of working. It is plausible that controlling for occupational status as we have done here is not a sufficient strategy to accommodate measurement issues of this kind. Receipt of SA tends also to increase during times of economic growth, which might reflect a moral hazard problem and thus variation in take-up behaviours [56]. Furthermore, retired persons are not eligible for SA, but in the ages analysed here they are few, or less than three percent of the study population [57]. The present findings apply also to a country with generous social security and labour force participation of both men and women and need not, therefore, be representative for dissimilar contexts.

Another limitation is that family fixed effect models cannot account for familial factors that are time-variant. They do not, therefore, capture that parents may treat children differently over time. Health behaviours may play a role on this concern, and register data typically lack such information. Associations between birth order and health outcomes may, thus, vary in strength over time and space [58], which we could not control for explicitly.

We had no data on diagnose-specific SA receipt before 2004, which means not before age 35 with the setup used for the analyses. We have used additional data on sickness absence without cause, from the years 1993–2003, to evaluate whether inclusion of an indicator for sickness absence before age 35 affect the results reported, and find that they do not. Results of these additional regressions are available from the authors upon request.

Finally, our analytical approach, using sibling fixed effects models, requires variation in the outcome, meaning that at least one sibling must have experienced the event that is studied, and singletons are not included into the regressions whatsoever. This implies that the study persons need not be representative for the population at large. Corresponding estimates from non-fixed regressions, that is, standard between-family models, generally display different estimates for the interrelation between birth order and health [9]. However, those would be based on comparisons of siblings across families, and families differ socially and genetically, which is a notorious problem from the perspective of residual confounding. The method of sibling fixed effects implies also reduced data size for the regressions on sibling groups; compare the numbers of SA recipients, sibling groups, siblings and person years between Tables 1 and 2. However, in the present case, data size remains sufficiently large even for the analyses of SA receipt by main cause.

To conclude, some evidence for a birth order pattern in working ages was observed, where first borns were the least likely to be sick because of mental or musculoskeletal disorders, and the associations were more accentuated for women than men. The results were obtained with sibling comparison models, which certifies that the estimated interrelations stem from variation within families and not from variation between families. The underlying mechanisms constitute an avenue for future research.

## Supporting information

S1 Table Hazard ratios with 95% confidence intervals for estimated associations between birth order and sickness absence- by sibling group size- results from Cox regression models stratified by shared mother and father identification. (DOCX)

S2 Table Hazard ratios with 95% confidence intervals for estimated associations between birth order and sickness absence, by birth spacing, results from Cox regression models stratified by shared mother and father identification. (DOCX)

## Abbreviations

CI: confidence interval
HR: hazard ratio
SA: sickness allowance
