# Supplementary material for: Birth order and sickness absence: Register-based evidence from Finland
Source: PLoS One. 2023 Jan 17;18(1):e0280532. doi: 10.1371/journal.pone.0280532 (PMC9844843; doi:10.1371/journal.pone.0280532)
Supplement: S1 Table — (DOCX) [file pone.0280532.s001.docx]

| Table S1. Hazard ratios with 95% confidence intervals for estimated associations between birth order and sickness absence- by sibling | | | | | | | | | | | | | | | | | | |
| --- | --- | --- | --- | --- | --- | --- | --- | --- | --- | --- | --- | --- | --- | --- | --- | --- | --- | --- |
| group size- results from Cox regression models stratified by shared mother and father identification | | | | | | | | | | | | | | |  |  |  |  |
|  |  |  |  |  |  |  |  |  |  |  |  |  |  |  |  |  |  |  |
|  |  |  |  |  |  |  |  |  |  |  |  |  |  |  |  |  |  |  |
|  |  | 2 siblings | | | | |  | 3 siblings | | | | |  | 4+ siblings | | | | |
|  |  |  |  |  |  |  |  |  |  |  |  |  |  |  |  |  |  |  |
|  |  |  |  |  |  |  |  |  |  |  |  |  |  |  |  |  |  |  |
|  |  | Model 1 | |  | Model 6 | |  | Model 1 | |  | Model 6 | |  | Model 1 | |  | Model 6 | |
| Cause of SA receipt |  |  |  |  |  |  |  |  |  |  |  |  |  |  |  |  |  |  |
| by birth order |  | HR | 95% CI |  | HR | 95% CI |  | HR | 95% CI |  | HR | 95% CI |  | HR | 95% CI |  | HR | 95% CI |
|  |  |  |  |  |  |  |  |  |  |  |  |  |  |  |  |  |  |  |
|  |  |  |  |  |  |  |  |  |  |  |  |  |  |  |  |  |  |  |
| All causes |  |  |  |  |  |  |  |  |  |  |  |  |  |  |  |  |  |  |
| 1st |  | 1 |  |  | 1 |  |  | 1 |  |  | 1 |  |  | 1 |  |  | 1 |  |
| 2nd |  | 1.05 | 1.02-1.08 | | 1.03 | 1.00-1.06 | | 1.00 | 0.96-1.04 | | 0.98 | 0.94-1.02 | | 1.07 | 0.98-1.17 | | 1.05 | 0.96-1.16 |
| 3rd |  |  |  |  |  |  |  | 1.00 | 0.94-1.08 | | 0.98 | 0.91-1.06 | | 0.99 | 0.86-1.13 | | 0.99 | 0.86-1.15 |
| 4th or higher |  |  |  |  |  |  |  |  |  |  |  |  |  | 1.06 | 0.88-1.27 | | 1.05 | 0.86-1.28 |
|  |  |  |  |  |  |  |  |  |  |  |  |  |  |  |  |  |  |  |
| Number of SA recipients | | 50,833 | | | | |  | 20,874 | | | | |  | 4,800 | | | | |
| Number of sibling groups | | 50,833 | | | | |  | 14,806 | | | | |  | 2,654 | | | | |
| Number of siblings |  | 101,666 | | | | |  | 44,418 | | | | |  | 10,670 | | | | |
| Number of person years | | 585,842 | | | | |  | 255,758 | | | | |  | 63,271 | | | | |
|  |  |  |  |  |  |  |  |  |  |  |  |  |  |  |  |  |  |  |
| Mental disorders |  |  |  |  |  |  |  |  |  |  |  |  |  |  |  |  |  |  |
| 1st |  | 1 |  |  | 1 |  |  | 1 |  |  | 1 |  |  | 1 |  |  | 1 |  |
| 2nd |  | 1.07 | 1.00-1.13 | | 1.03 | 0.97-1.10 | | 0.93 | 0.84-1.02 | | 0.89 | 0.81-0.99 | | 1.14 | 0.93-1.39 | | 1.14 | 0.91-1.41 |
| 3rd |  |  |  |  |  |  |  | 1.01 | 0.86-1.19 | | 0.97 | 0.81-1.16 | | 1.13 | 0.83-1.56 | | 1.17 | 0.83-1.66 |
| 4th or higher |  |  |  |  |  |  |  |  |  |  |  |  |  | 1.73 | 1.14-2.63 | | 1.89 | 1.20-2.97 |
|  |  |  |  |  |  |  |  |  |  |  |  |  |  |  |  |  |  |  |
| Number of SA recipients | | 15,717 | | | | |  | 4,932 | | | | |  | 1,070 | | | | |
| Number of sibling groups | | 15,717 | | | | |  | 4,499 | | | | |  | 911 | | | | |
| Number of siblings |  | 31,434 | | | | |  | 13,497 | | | | |  | 3,671 | | | | |
| Number of person years | | 153,015 | | | | |  | 69,141 | | | | |  | 20,307 | | | | |
|  |  |  |  |  |  |  |  |  |  |  |  |  |  |  |  |  |  |  |
| Musculoskeletal disorders | | |  |  |  |  |  |  |  |  |  |  |  |  |  |  |  |  |
| 1st |  | 1 |  |  | 1 |  |  | 1 |  |  | 1 |  |  | 1 |  |  | 1 |  |
| 2nd |  | 1.12 | 1.07-1.18 | | 1.09 | 1.03-1.16 | | 1.10 | 1.01-1.19 | | 1.04 | 0.95-1.14 | | 1.16 | 0.97-1.38 | | 1.12 | 0.92-1.35 |
| 3rd |  |  |  |  |  |  |  | 1.18 | 1.02-1.37 | | 1.08 | 0.93-1.27 | | 1.08 | 0.82-1.41 | | 1.07 | 0.80-1.44 |
| 4th or higher |  |  |  |  |  |  |  |  |  |  |  |  |  | 1.18 | 0.82-1.71 | | 1.19 | 0.79-1.78 |
|  |  |  |  |  |  |  |  |  |  |  |  |  |  |  |  |  |  |  |
| Number of SA recipients | | 19,062 | | | | |  | 6,286 | | | | |  | 1,335 | | | | |
| Number of sibling groups | | 19,062 | | | | |  | 5,553 | | | | |  | 1,111 | | | | |
| Number of siblings |  | 38,124 | | | | |  | 16,659 | | | | |  | 4,473 | | | | |
| Number of person years | | 187,774 | | | | |  | 84,741 | | | | |  | 24,171 | | | | |
|  |  |  |  |  |  |  |  |  |  |  |  |  |  |  |  |  |  |  |
| Injuries |  |  |  |  |  |  |  |  |  |  |  |  |  |  |  |  |  |  |
| 1st |  | 1 |  |  | 1 |  |  | 1 |  |  | 1 |  |  | 1 |  |  | 1 |  |
| 2nd |  | 1.07 | 1.01-1.14 | | 1.06 | 1.00-1.14 | | 1.08 | 0.97-1.19 | | 1.07 | 0.96-1.19 | | 0.99 | 0.80-1.22 | | 0.97 | 0.77-1.23 |
| 3rd |  |  |  |  |  |  |  | 1.09 | 0.92-1.29 | | 1.08 | 0.90-1.30 | | 0.91 | 0.66-1.26 | | 0.91 | 0.64-1.31 |
| 4th or higher |  |  |  |  |  |  |  |  |  |  |  |  |  | 0.83 | 0.53-1.30 | | 0.83 | 0.51-1.35 |
|  |  |  |  |  |  |  |  |  |  |  |  |  |  |  |  |  |  |  |
| Number of SA recipients | | 15,220 | | | | |  | 4,657 | | | | |  | 965 | | | | |
| Number of sibling groups | | 15,220 | | | | |  | 4,291 | | | | |  | 841 | | | | |
| Number of siblings |  | 30,440 | | | | |  | 12,873 | | | | |  | 3,393 | | | | |
| Number of person years | | 152,197 | | | | |  | 67,844 | | | | |  | 18,596 | | | | |
|  |  |  |  |  |  |  |  |  |  |  |  |  |  |  |  |  |  |  |
| Other causes |  |  |  |  |  |  |  |  |  |  |  |  |  |  |  |  |  |  |
| 1st |  | 1 |  |  | 1 |  |  | 1 |  |  | 1 |  |  | 1 |  |  | 1 |  |
| 2nd |  | 0.99 | 0.95-1.03 | | 0.98 | 0.93-1.02 | | 0.93 | 0.87-0.99 | | 0.93 | 0.86-0.99 | | 0.99 | 0.86-1.14 | | 0.97 | 0.83-1.13 |
| 3rd |  |  |  |  |  |  |  | 0.85 | 0.76-0.96 | | 0.86 | 0.76-0.97 | | 0.90 | 0.72-1.12 | | 0.90 | 0.71-1.14 |
| 4th or higher |  |  |  |  |  |  |  |  |  |  |  |  |  | 0.84 | 0.62-1.13 | | 0.84 | 0.60-1.16 |
|  |  |  |  |  |  |  |  |  |  |  |  |  |  |  |  |  |  |  |
| Number of SA recipients | | 29,164 | | | | |  | 9,769 | | | | |  | 2,013 | | | | |
| Number of sibling groups | | 29,164 | | | | |  | 8,390 | | | | |  | 1,587 | | | | |
| Number of siblings |  | 58,328 | | | | |  | 25,170 | | | | |  | 6,452 | | | | |
| Number of person years | | 298,405 | | | | |  | 136,046 | | | | |  | 36,307 | | | | |
|  |  |  |  |  |  |  |  |  |  |  |  |  |  |  |  |  |  |  |
|  |  |  |  |  |  |  |  |  |  |  |  |  |  |  |  |  |  |  |
| Model 1 adjusts for Birth order, Sex and Birth year. Model 6 adjusts for Birth order, Sex, Birth year, Motherʾs age at birth, Educational | | | | | | | | | | | | | | | | | | |
| Level, Occupation, Income quintile and Family composition. | | | | | | | | |  |  |  |  |  |  |  |  |  |  |
