# Supplementary material for: Birth order and sickness absence: Register-based evidence from Finland
Source: PLoS One. 2023 Jan 17;18(1):e0280532. doi: 10.1371/journal.pone.0280532 (PMC9844843; doi:10.1371/journal.pone.0280532)
Supplement: S2 Table — (DOCX) [file pone.0280532.s002.docx]

| Table S2. Hazard ratios with 95% confidence intervals for estimated associations between birth order and sickness absence, by birth | | | | | | | | | | | | | | | | | | |
| --- | --- | --- | --- | --- | --- | --- | --- | --- | --- | --- | --- | --- | --- | --- | --- | --- | --- | --- |
| spacing, results from Cox regression models stratified by shared mother and father identification | | | | | | | | | | | | | | |  |  |  |  |
|  |  |  |  |  |  |  |  |  |  |  |  |  |  |  |  |  |  |  |
|  |  |  |  |  |  |  |  |  |  |  |  |  |  |  |  |  |  |  |
|  |  | At most 2 years | | | | |  | 3 years | | | | |  | 4+ years | | | | |
|  |  |  |  |  |  |  |  |  |  |  |  |  |  |  |  |  |  |  |
|  |  |  |  |  |  |  |  |  |  |  |  |  |  |  |  |  |  |  |
|  |  | Model 1 | |  | Model 6 | |  | Model 1 | |  | Model 6 | |  | Model 1 | |  | Model 6 | |
| Cause of SA receipt |  |  |  |  |  |  |  |  |  |  |  |  |  |  |  |  |  |  |
| by birth order |  | HR | 95% CI |  | HR | 95% CI |  | HR | 95% CI |  | HR | 95% CI |  | HR | 95% CI |  | HR | 95% CI |
|  |  |  |  |  |  |  |  |  |  |  |  |  |  |  |  |  |  |  |
|  |  |  |  |  |  |  |  |  |  |  |  |  |  |  |  |  |  |  |
| All causes |  |  |  |  |  |  |  |  |  |  |  |  |  |  |  |  |  |  |
| 1st |  | 1 |  |  | 1 |  |  | 1 |  |  | 1 |  |  | 1 |  |  | 1 |  |
| 2nd |  | 1.04 | 1.00-1.07 | | 1.01 | 0.98-1.05 | | 1.02 | 0.96-1.08 | | 0.99 | 0.93-1.06 | | 1.01 | 0.97-1.05 | | 1.00 | 0.95-1.04 |
| 3rd |  | 1.00 | 0.92-1.09 | | 0.98 | 0.89-1.07 | | 1.03 | 0.92-1.15 | | 0.99 | 0.87-1.12 | | 1.03 | 0.96-1.11 | | 1.02 | 0.94-1.10 |
| 4th or higher |  | 1.01 | 0.82-1.23 | | 0.97 | 0.78-1.19 | | 0.90 | 0.73-1.12 | | 0.88 | 0.70-1.10 | | 1.09 | 0.96-1.24 | | 1.05 | 0.92-1.20 |
|  |  |  |  |  |  |  |  |  |  |  |  |  |  |  |  |  |  |  |
| Number of SA recipients | | 20,060 | | | | |  | 16,834 | | | | |  | 39,613 | | | | |
| Number of sibling groups | | 18,911 | | | | |  | 15,143 | | | | |  | 34,239 | | | | |
| Number of siblings |  | 40,609 | | | | |  | 34,578 | | | | |  | 81,567 | | | | |
| Number of person years | | 245,502 | | | | |  | 201,668 | | | | |  | 457,701 | | | | |
|  |  |  |  |  |  |  |  |  |  |  |  |  |  |  |  |  |  |  |
| Mental disorders |  |  |  |  |  |  |  |  |  |  |  |  |  |  |  |  |  |  |
| 1st |  | 1 |  |  | 1 |  |  | 1 |  |  | 1 |  |  | 1 |  |  | 1 |  |
| 2nd |  | 1.06 | 0.99-1.15 | | 1.00 | 0.92-1.09 | | 1.08 | 0.94-1.24 | | 1.07 | 0.92-1.26 | | 1.03 | 0.94-1.13 | | 1.03 | 0.93-1.14 |
| 3rd |  | 0.89 | 0.73-1.09 | | 0.81 | 0.65-1.01 | | 1.45 | 1.11-1.89 | | 1.44 | 1.07-1.93 | | 1.09 | 0.93-1.29 | | 1.10 | 0.93-1.31 |
| 4th or higher |  | 1.47 | 0.95-2.27 | | 1.36 | 0.87-2.14 | | 1.72 | 1.06-2.78 | | 1.80 | 1.07-3.01 | | 1.60 | 1.21-2.12 | | 1.60 | 1.19-2.14 |
|  |  |  |  |  |  |  |  |  |  |  |  |  |  |  |  |  |  |  |
| Number of SA recipients | | 6,219 | | | | |  | 4,835 | | | | |  | 10,665 | | | | |
| Number of sibling groups | | 6,132 | | | | |  | 4,713 | | | | |  | 10,282 | | | | |
| Number of siblings |  | 13,166 | | | | |  | 10,792 | | | | |  | 24,644 | | | | |
| Number of person years | | 67,080 | | | | |  | 53,987 | | | | |  | 121,396 | | | | |
|  |  |  |  |  |  |  |  |  |  |  |  |  |  |  |  |  |  |  |
| Musculoskeletal disorders | | |  |  |  |  |  |  |  |  |  |  |  |  |  |  |  |  |
| 1st |  | 1 |  |  | 1 |  |  | 1 |  |  | 1 |  |  | 1 |  |  | 1 |  |
| 2nd |  | 1.10 | 1.04-1.18 | | 1.09 | 1.01-1.17 | | 1.09 | 0.97-1.22 | | 1.06 | 0.93-1.22 | | 1.07 | 0.99-1.16 | | 1.01 | 0.93-1.11 |
| 3rd |  | 1.21 | 1.02-1.43 | | 1.19 | 0.99-1.43 | | 1.07 | 0.85-1.34 | | 1.05 | 0.82-1.36 | | 1.14 | 0.99-1.31 | | 1.05 | 0.90-1.22 |
| 4th or higher |  | 0.93 | 0.61-1.42 | | 0.97 | 0.62-1.50 | | 0.97 | 0.63-1.48 | | 0.97 | 0.61-1.54 | | 1.17 | 0.90-1.53 | | 1.05 | 0.79-1.38 |
|  |  |  |  |  |  |  |  |  |  |  |  |  |  |  |  |  |  |  |
| Number of SA recipients | | 7,564 | | | | |  | 5,866 | | | | |  | 13,253 | | | | |
| Number of sibling groups | | 7,414 | | | | |  | 5,673 | | | | |  | 12,639 | | | | |
| Number of siblings |  | 15,956 | | | | |  | 13,008 | | | | |  | 30,292 | | | | |
| Number of person years | | 82,817 | | | | |  | 65,922 | | | | |  | 147,947 | | | | |
|  |  |  |  |  |  |  |  |  |  |  |  |  |  |  |  |  |  |  |
| Injuries |  |  |  |  |  |  |  |  |  |  |  |  |  |  |  |  |  |  |
| 1st |  | 1 |  |  | 1 |  |  | 1 |  |  | 1 |  |  | 1 |  |  | 1 |  |
| 2nd |  | 1.03 | 0.96-1.11 | | 1.04 | 0.95-1.13 | | 1.07 | 0.93-1.23 | | 1.04 | 0.90-1.23 | | 1.08 | 0.98-1.19 | | 1.09 | 0.98-1.21 |
| 3rd |  | 0.98 | 0.91-1.20 | | 1.02 | 0.82-1.26 | | 1.07 | 0.82-1.41 | | 1.08 | 0.80-1.47 | | 1.15 | 0.97-1.37 | | 1.16 | 0.96-1.39 |
| 4th or higher |  | 0.88 | 0.55-1.40 | | 0.93 | 0.57-1.50 | | 0.70 | 0.40-1.21 | | 0.71 | 0.40-1.29 | | 1.13 | 0.82-1.54 | | 1.11 | 0.80-1.53 |
|  |  |  |  |  |  |  |  |  |  |  |  |  |  |  |  |  |  |  |
| Number of SA recipients | | 5,962 | | | | |  | 4,683 | | | | |  | 10,197 | | | | |
| Number of sibling groups | | 5,880 | | | | |  | 4,581 | | | | |  | 9,891 | | | | |
| Number of siblings |  | 12,620 | | | | |  | 10,414 | | | | |  | 23,672 | | | | |
| Number of person years | | 66,096 | | | | |  | 53,516 | | | | |  | 119,025 | | | | |
|  |  |  |  |  |  |  |  |  |  |  |  |  |  |  |  |  |  |  |
| Other causes |  |  |  |  |  |  |  |  |  |  |  |  |  |  |  |  |  |  |
| 1st |  | 1 |  |  | 1 |  |  | 1 |  |  | 1 |  |  | 1 |  |  | 1 |  |
| 2nd |  | 0.97 | 0.92-1.03 | | 0.96 | 0.90-1.02 | | 0.94 | 0.85-1.04 | | 0.89 | 0.80-1.00 | | 0.92 | 0.87-0.99 | | 0.93 | 0.87-1.00 |
| 3rd |  | 0.92 | 0.80-1.06 | | 0.88 | 0.76-1.03 | | 0.83 | 0.69-1.00 | | 0.77 | 0.63-0.95 | | 0.90 | 0.80-1.01 | | 0.91 | 0.80-1.03 |
| 4th or higher |  | 0.90 | 0.64-1.28 | | 0.84 | 0.59-1.20 | | 0.73 | 0.52-1.03 | | 0.67 | 0.46-0.96 | | 0.81 | 0.65-1.00 | | 0.81 | 0.65-1.01 |
|  |  |  |  |  |  |  |  |  |  |  |  |  |  |  |  |  |  |  |
| Number of SA recipients | | 11,184 | | | | |  | 9,235 | | | | |  | 20,527 | | | | |
| Number of sibling groups | | 10,937 | | | | |  | 8,842 | | | | |  | 19,362 | | | | |
| Number of siblings |  | 23,525 | | | | |  | 20,238 | | | | |  | 46,187 | | | | |
| Number of person years | | 127,120 | | | | |  | 106,453 | | | | |  | 237,186 | | | | |
|  |  |  |  |  |  |  |  |  |  |  |  |  |  |  |  |  |  |  |
|  |  |  |  |  |  |  |  |  |  |  |  |  |  |  |  |  |  |  |
| Model 1 adjusts for Birth order, Sex and Birth year. Model 6 adjusts for Birth order, Sex, Birth year, Motherʾs age at birth, Educational | | | | | | | | | | | | | | | | | | |
| level, Occupation, Income quintile and Family composition. | | | | | | | | |  |  |  |  |  |  |  |  |  |  |
| Birth spacing refers to the age difference between any two siblings in a sibling group. | | | | | | | | | | | | |  |  |  |  |  |  |
